# Effectiveness of tuning an artificial intelligence algorithm for cerebral aneurysm diagnosis: a study of 10,000 consecutive cases

**DOI:** 10.1038/s41598-023-43418-x

**Published:** 2023-09-27

**Authors:** Masashi Kuwabara, Fusao Ikawa, Shigeyuki Sakamoto, Takahito Okazaki, Daizo Ishii, Masahiro Hosogai, Yuyo Maeda, Masaaki Chiku, Naoyuki Kitamura, Antoine Choppin, Daisaku Takamiya, Yuki Shimahara, Takeo Nakayama, Kaoru Kurisu, Nobutaka Horie

**Affiliations:** 1 Department of Neurosurgery, Graduate School of Biomedical and Health Sciences, Hiroshima University, 1-2-3 Kasumi, Minami-ku, Hiroshima, Hiroshima 734-8551 Japan; 2 Department of Neurosurgery, Shimane Prefectural Central Hospital, 4-1-1 Himebara, Izumo, Shimane 693-8555 Japan; 3 Department of Neurosurgery, Medical Check Studio, Tokyo Ginza Clinic, 1-2-4 Ginza, Chuo-ku, Tokyo, 104-0061 Japan; 4 Department of Diagnostic Radiology, Kasumi Clinic, 1-2-27 Shinonomehommachi, Minami-ku, Hiroshima, Hiroshima 734-0023 Japan; 5 LPIXEL Inc., 1-6-1 Otemachi, Chiyoda-ku, Tokyo, 100-0004 Japan; 6 Department of Health Informatics, School of Public Health, Graduate School of Medicine, Kyoto University, Yoshida-Konoe, Sakyo-ku, Kyoto, Kyoto 606-8501 Japan; 7 Chugoku Rosai Hospital, 1-5-1 Hirotagaya, Kure, Hiroshima 737-0193 Japan

**Keywords:** Stroke, Brain

## Abstract

Diagnostic image analysis for unruptured cerebral aneurysms using artificial intelligence has a very high sensitivity. However, further improvement is needed because of a relatively high number of false positives. This study aimed to confirm the clinical utility of tuning an artificial intelligence algorithm for cerebral aneurysm diagnosis. We extracted 10,000 magnetic resonance imaging scans of participants who underwent brain screening using the “Brain Dock” system. The sensitivity and false positives/case for aneurysm detection were compared before and after tuning the algorithm. The initial diagnosis included only cases for which feedback to the algorithm was provided. In the primary analysis, the sensitivity of aneurysm diagnosis decreased from 96.5 to 90% and the false positives/case improved from 2.06 to 0.99 after tuning the algorithm (*P* < 0.001). In the secondary analysis, the sensitivity of aneurysm diagnosis decreased from 98.8 to 94.6% and the false positives/case improved from 1.99 to 1.03 after tuning the algorithm (*P* < 0.001). The false positives/case reduced without a significant decrease in sensitivity. Using large clinical datasets, we demonstrated that by tuning the algorithm, we could significantly reduce false positives with a minimal decline in sensitivity.

## Introduction

The prevalence of asymptomatic unruptured cerebral aneurysms (UCAs) in adults ranges from 2 to 6%^1–3^, with a rupture rate of 0.95% per year in Japan^4^. The risk of rupture of a UCA in Japanese individuals is reportedly 2.8 times higher than that in Westerners, which emphasizes the importance of early detection of UCAs by brain screening in Japan^5,6^. The Brain Dock is a screening system for examining the brain, and is supported by some organizations and municipalities and is widely available in Japan. The Brain Dock system detects asymptomatic cerebral infarction, UCAs, and brain tumors using magnetic resonance imaging (MRI) and magnetic resonance angiography (MRA) at an early stage to enable the treatment of healthy participants, with an effort toward preventing stroke and dementia^5,7,8^. Another advantage of the Brain Dock is that it allows the patient to review his or her lifestyle, and any risk factors detected will provide an opportunity to reduce the risk of cerebrovascular disorders in the future by improving lifestyle. In other words, the Brain Dock is a unique Japanese preventive medicine that aims at early detection and prevention^5^. Currently, evaluation using the Brain Dock is double-checked by diagnostic imaging physicians whose tasks are to review an enormous number of images to confirm the diagnosis. With the development of cloud-based communication technology, however, a brain diagnosis system that integrates cloud computing and artificial intelligence (AI)-based remote image diagnosis has been introduced as a less time-consuming approach.

The AI-based UCA imaging software used in this study has attracted attention due to its high diagnostic sensitivity^9–15^. Although its sensitivity is high, the use of the software in clinical practice produces a certain number of false positives (FPs), which increases the workload for diagnostic imaging physicians. Thus, further improvement is required for clinical application.

This study aimed to investigate the effects of tuning an AI algorithm for UCA diagnosis on the sensitivity and FPs/case of UCA detection based on large-scale brain scan data from 10,000 consecutive cases acquired using the Brain Dock system.

## Results

Of the 10,000 Brain Dock images, we compared 5000 images that were analyzed prior to tuning the AI algorithm with a second set of 5000 images that were analyzed after tuning (Table 1). The average age of participants analyzed with the initial AI algorithm was less than that of those analyzed after tuning. There was no significant difference in the sample sex ratio (percentage of women) or body mass index (BMI) prior to versus after tuning. Further, no significant differences were observed in the medical history of the participants between the two groups.

**Table 1:** Baseline characteristics of patients in the primary analysis.

Characteristics	Total (N = 10,000)	Before tuning (n = 5,000)	After tuning (n = 5,000)	*P-*value
Age, years	48.5 ± 11.6	47.6 ± 11.5	49.4 ± 11.6	< 0.001*
Sex (female)	4345 (43.5%)	2153 (43.1%)	2192 (43.8%)	0.443
BMI	23.1 ± 3.52	23.0 ± 3.48	23.1 ± 3.56	0.078
Medical history				
Hypertension	1706 (17.1%)	812 (16.2%)	894 (17.9%)	0.067
Diabetes mellitus	395 (4.0%)	187 (3.7%)	208 (4.2%)	0.504
Dyslipidemia	1670 (16.7%)	827 (16.5%)	843 (16.9%)	0.379
Arrhythmia	573(5.7%)	290 (5.8%)	283 (5.7%)	0.761
Stroke	34 (0.34%)	13 (0.26%)	21 (0.42%)	0.389
Dementia	8 (0.08%)	3 (0.06%)	5 (0.1%)	0.843
Surgery	3316 (33.2%)	1615 (32.3%)	1701 (34.0%)	0.184
Cerebral aneurysm				< 0.001*
None	8962 (89.6%)	4422 (88.4%)	4540 (90.8%)	
Suspicion	838 (8.4%)	478 (9.6%)	360 (7.2%)	
Definite	200 (2.0%)	100 (2.0%)	100 (2.0%)	

Data are represented as the mean ± standard deviation or n (%).

*BMI* body mass index.

*P < 0.05.

We detected 4422/5000 (88.4%) cases without UCA, 100/5000 (2.0%) with UCA, and 478/5000 (9.6%) with suspected UCA using the initial AI algorithm. In contrast, after tuning, we detected 4540/5000 (90.8%) cases without UCA, 100/5000 (2.0%) with UCA, and 360/5000 (7.2%) with suspected UCA. More cases without UCA were observed after tuning the algorithm than before, with a statistically significant difference (*P* < 0.001).

For the 2218 feedback cases, we compared the cases analyzed before (1359 cases) and after (859 cases) tuning the algorithm (Table 2). In the secondary analysis, the average age of participants whose data were analyzed before tuning was less than that of participants whose data were analyzed after tuning (48.5 ± 11.8 vs. 50.3 ± 11.6 years, respectively; *P* < 0.001). The percentage of women in the group analyzed before the tuning was lower than that in the group analyzed after tuning (592/1359 [43.6%] vs. 423/859 [49.2%], respectively; *P* = 0.009). There was no statistically significant difference in BMI between the groups (22.9 ± 3.44 before versus 22.9 ± 3.54 after).

**Table 2:** Baseline characteristics of patients in the secondary analysis.

Characteristics	Total (n = 2218)	Before tuning (n = 1359)	After tuning (n = 859)	*P-*value
Age	49.2 ± 11.8	48.5 ± 11.8	50.3 ± 11.6	< 0.001*
Sex (female)	1015 (45.8%)	592 (43.6%)	423 (49.2%)	0.009*
BMI	22.9 ± 3.48	22.9 ± 3.44	22.9 ± 3.54	0.863
Medical history				
Hypertension	386 (17.4%)	224 (16.5%)	162 (18.9%)	0.355
Diabetes mellitus	97 (4.4%)	56 (4.1%)	41 (4.8%)	0.009*
Dyslipidemia	370 (16.7%)	215 (15.8%)	155 (18.0%)	0.394
Arrhythmia	133 (6.0%)	76 (5.6%)	57 (6.6%)	0.572
Stroke	6 (0.3%)	3 (0.2%)	3 (0.3%)	0.441
Dementia	0 (0%)	0 (0%)	0 (0%)	1.000
Surgery	753 (33.9%)	457 (33.6%)	296 (34.5%)	0.041*
Cerebral aneurysm				0.681
None	1919 (86.5%)	1182 (87.0%)	737 (85.8%)	
Suspicion	247 (11.1%)	145 (10.6%)	102 (11.9%)	
Definite	52 (2.3%)	32 (2.4%)	20 (2.3%)	

Data are represented as the mean ± standard deviation or n (%).

*BMI* body mass index.

*P < 0.05.

No significant differences were observed when comparing hypertension, dyslipidemia, arrhythmia, stroke, and dementia between the two groups. However, the occurrence of diabetes was lower in the group analyzed before tuning the AI algorithm than in the group analyzed after tuning (56/1359 [4.1%] vs. 41/859 [4.8%], respectively; *P* = 0.009). Furthermore, fewer participants in the group analyzed before tuning had a surgical history than those in the group analyzed after tuning (457/1359 [33.6%] vs. 296/859 [34.5%], respectively; *P* = 0.041). No significant difference was observed in the UCA detection rate before tuning versus after tuning.

Table 3 shows the results of the accuracy analysis performed for diagnoses obtained before and after tuning the diagnosis algorithm (primary analysis). For the 5000 cases analyzed before tuning, the sensitivity of UCA diagnosis was 96.5% and the FPs/case was 2.06. In contrast, for the 5000 cases analyzed after tuning the AI algorithm, the sensitivity of UCA diagnosis was 90.0% (*P* < 0.01) and the FPs/case was 0.99 (*P* < 0.001).

**Table 3:** Primary analysis of the accuracy of UCA diagnosis (n = 10,000).

Before tuning (n = 5000)			After tuning (n = 5000)		
	AI (P)	AI (N)		AI (P)	AI (N)
Physician (P)	558/5000	20/5000	Physician (P)	414/5000	46/5000
Physician (N)	3777/5000	645/5000	Physician (N)	2617/5000	1923/5000
Sensitivity (%)	96.5			90.0	
FPs/case	2.06			0.99	

*AI* artificial intelligence, *FP* false positive, *N* negative, *P* positive, *UCA* unruptured cerebral aneurysm.

Table 4 shows the secondary analysis of diagnostic accuracy for detecting UCA. For the 1359 participants analyzed before tuning the AI algorithm, the sensitivity was 98.8% and the FPs/case was 1.99. In contrast, for the 859 cases analyzed after tuning the algorithm, the sensitivity was 94.6% (*P* = 0.05) and the FPs/case was 1.03 (*P* < 0.001).

**Table 4:** Secondary analysis of the accuracy of UCA diagnosis (n = 2218).

Before tuning (n = 1359)			After tuning (n = 859)		
	AI (P)	AI (N)		AI (P)	AI (N)
Physician (P)	163/1359	2/1359	Physician (P)	106/859	6/859
Physician (N)	1064/1359	130/1359	Physician (N)	480/859	267/859
Sensitivity (%)	98.8			94.6	
FPs/case	1.99			1.03	

*AI* artificial intelligence, *FP* false positive, *N* negative, *P* positive, *UCA* unruptured cerebral aneurysm.

Figure 4a shows the distribution of cases for each number of aneurysm candidates in the primary analysis. Figure 4b shows the rate of cases for each number of FPs in the secondary analysis. We observed that the rate of cases with 0 or 1 FP was the highest after tuning the AI algorithm and the rate of cases with 4 or 5 FPs was negligible.

Using data from 2218 participants, we analyzed the relationship between sensitivity and FPs/case gained by changing the maximum number of candidate output points and found that the FPs/case was significantly reduced by tuning the algorithm, accompanied by a minimal decline in sensitivity (Fig. 5 and Table 5).

**Table 5:** Coordinates of FPs/case and sensitivity before and after tuning the AI algorithm.

Before tuning		After tuning	
FPs/case	Sensitivity	FPs/case	Sensitivity
0	0	0	0
0.81	76.4%	0.58	81.3%
1.43	87.3%	0.88	91.1%
1.77	94.5%	0.99	94.6%
1.92	97.0%	1.02	94.6%
1.99	98.8%	1.03	94.6%

*AI* artificial intelligence, *FP* false positive.

## Discussion

By analyzing the relationship between sensitivity and FPs/case gained by changing the maximum number of candidate output points, the FPs/case decreased significantly with a minimal decline in sensitivity after the AI algorithm was tuned. The clinical utility of AI-assisted UCA diagnosis was thus confirmed in 10,000 consecutive cases. In this study, participants underwent brain screening using the Brain Dock system. The Brain Dock is a preventive medicine unique to Japan, aiming at early detection and prevention of asymptomatic brain diseases. Despite a high FP rate, prevention of cardiovascular diseases is reportedly more important than treatment^16,17^. Considering that cerebrovascular diseases result in serious sequelae or even death in Japan with a higher incidence than in other countries, Brain Docks are a promising means of screening that can detect risks at an early stage and lead to prevention.

Over the past few years, AI-based UCA diagnosis using deep learning has rapidly become a promising tool^11–14^. In a similar deep learning-based UCA diagnosis system, the number of UCAs ranged from 31 to 551 cases, the sensitivity ranged from 70 to 100%, the FPs/case ranged from 2.9 to 10.0, and the UCA detection rate increased by 4.8–12.5% when diagnosed with the aid of AI^9,12,18–21^. Although highly sensitive, the diagnosis of UCAs using AI algorithms still needs improvement for routine clinical applications due to the relatively high FP rate^9,12,19–21^. This high number of FPs limits the clinical utility of AI-based UCA diagnosis because diagnostic imaging physicians remain skeptical about the results of AI and interpreting the images is time-consuming. In contrast, the number of cases in this study was 10,000 as a whole, and by tuning the AI algorithm, the number of FPs/case was significantly reduced compared to that in previous reports. Therefore, it can be concluded that this study is a further development of the previous reports.

The diagnostic threshold value of the AI system was tuned on a separate external dataset from that used in this study. The sensitivity of the algorithm was high at a threshold of 0.5, but the high FPs/case limited its clinical application. When the threshold was progressively increased from 0.5 in increments of 0.1, the candidate threshold values that optimized both sensitivity and specificity were narrowed down to 0.8 or 0.9. However, a threshold of 0.9 showed a significant decrease in sensitivity for a group of cases with a large voxel size; therefore, a threshold of 0.8 was chosen, which provided better stability of results. This decrease in sensitivity had minimal negative clinical impact, and the reduction in FPs/case made the AI algorithm easier to use in clinical practice.

Given that the final clustering process is done after removing key points with low scores, increasing the cut-off threshold not only suppresses the number of final outputs but also removes noisy key points from each cluster, resulting in more precise coordinates of the final outputs. This means that the sensitivity and precision in regard to the cut-off threshold are not necessarily in a trade-off relationship as is often the case with a simpler detection pipeline. Although this was not the case in this report, it is theoretically possible that both metrics may improve at the same time.

We found that the diagnostic sensitivity for UCA changed from 98.8 to 94.6% and the FPs/case changed from 1.986 to 1.034, thereby improving the diagnostics to a clinically acceptable level. According to a systematic review, the sensitivity of diagnosis by board-certified neuroradiologists is reported to be 87–92%^22^, not 100%, which is comparable to that of AI-based diagnosis. Thus, a reduction in the sensitivity of AI-based diagnosis may not be a significant problem. Accordingly, analyzing these results enabled us to visualize a significant reduction in FPs/case with a tolerable reduction in sensitivity after tuning the algorithm (Fig. 5). If the FPs/case is reduced, the burden on the diagnostic imaging physician tasked with verifying the site of the UCA diagnosed by AI will also be reduced, thereby making the AI approach more clinically feasible. Furthermore, at this level of capability, the number of diagnostic imaging physicians required may be reduced from two to one because some primary imaging physicians could then be replaced by an AI assistant for the purpose of UCA diagnosis^9,12,19–21^.

The current Brain Dock in Japan is read by several neuroradiologists or neurosurgeons, each of whom has to read a huge amount of information, which is physically and time-consuming. If the AI reading is sufficiently reliable, it will not only maximize the preventive effect on participants undergoing the Brain Dock but also reduce the burden on the diagnostic imaging physicians involved in the Brain Dock. This study demonstrates the clinical utility of AI for UCA diagnosis, which is a promising step forward for neurosurgical practice in Japan. The advantages of AI diagnosis include low cost and time, as well as the ability to detect aneurysms in unusual locations that are easy to miss by physicians^12^. Alternatively, the disadvantages of AI are a reduction in opportunities or educational experiences in diagnosing UCAs for young physicians and some aneurysms could be missed due to signal loss caused by turbulence within a large aneurysm and internal thrombosis^12^; however, the latter can be tuned by retraining the AI algorithm.

This study has some limitations. First, we involved approximately 20 diagnostic imaging physicians to interpret the AI analysis. Therefore, diagnostic bias may be present due to differences between individual physicians. However, all physicians were board-certified radiologists of the Japan Radiological Society or board-certified neurosurgeons of the Japan Neurosurgical Society with more than 10 years of experience. Second, this was not a planned randomized controlled trial but a single-center retrospective observational study with potential biases. Third, since the evaluation was conducted using different datasets before and after threshold tuning, we could not completely correct the selection bias of the dataset. However, in the secondary analysis, there was no significant difference in the diagnosis of aneurysms by MRI. Therefore, the effect of this bias is likely small. There was also a significant difference in the mean age of the participants among the data sets, which could have affected the results. Fourth, cerebral angiography is the gold standard for the diagnosis of UCAs; however, cerebral angiography is invasive and possesses a risk of allergic reaction due to the use of contrast media. We believe the significance of this study is the contribution of diagnosing UCA based on tuning an AI algorithm in a large number of cases.

In conclusion, we demonstrated that by using large clinical datasets and tuning an AI algorithm, we could significantly reduce FPs/case with a minimal decline in sensitivity. The clinical utility of AI-assisted UCA diagnosis was thus confirmed in 10,000 consecutive cases. Understanding and using the properties of AI may reduce the physical and time burdens on diagnostic imaging physicians by further improving the accuracy of UCA diagnosis.

## Methods

### Ethics statement

This research was performed in accordance with the principles of the Declaration of Helsinki and meets the requirements of medical ethics. The Institutional Review Board of the Ethical Committee for Epidemiology of Hiroshima University approved this study (approval number E-1762). As individual data were anonymized and collected during routine Brain Dock examination, the requirement for obtaining individual informed consent was waived by the Institutional Review Board of the Ethical Committee for Epidemiology of Hiroshima University. This study was registered with the University Hospital Medical Information Network (ID: UMIN000043024, No: R000049102).

### Study design

Considering soundness and applicability, we adopted a published list of criteria for evaluating AI research^23^. We analyzed images acquired by the Brain Dock using the remote imaging system “LOOKREC” developed by Medical Network Systems Incorporated (MNES Inc., Hiroshima, Japan). Specifically, MRI and MRA of the heads of participants who underwent Brain Dock examinations at a single center were first uploaded to the cloud. This study was performed using one type of MRI system (Vantage Elan, 1.5T, Canon, Tokyo, Japan) equipped with a 32-channel head coil and a created integration with the viewer for the routine work of diagnostic imaging physicians. The AI-based UCA diagnosis software, trained using deep learning, was provided by LPIXEL Inc.^12^. The initial diagnosis was made by radiologists at MNES Inc. without using the AI-based software because of the absence of prior regulatory approval; however, the final diagnosis could be confirmed after reviewing the AI-based diagnosis. Further, neurosurgeons working at three university hospitals in Japan uploaded their final diagnostic results to the cloud. The final diagnosis was determined by reviewing these diagnostic results.

The diagnostic results were available on the cloud, allowing participants to access the final diagnosis while simultaneously allowing us to create a database. All scans were obtained using a 1.5 Tesla MRI system. Neuroimaging protocols included the following sequences: T1-weighted, T2-weighted, T2*-weighted, fluid-attenuated inversion recovery, diffusion-weighted image, head MRA, and neck MRA.

### Patient enrollment

**Figure 1:**
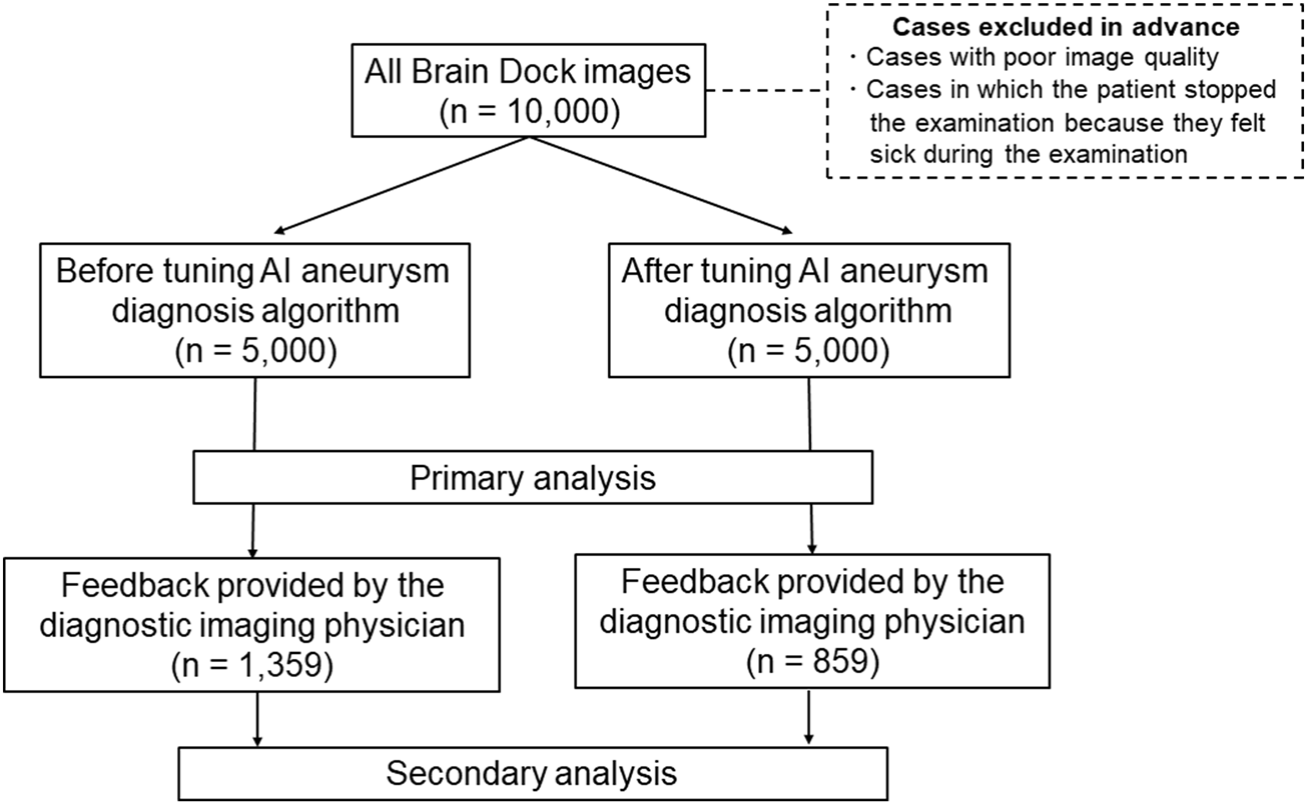
Flowchart of the process of patient inclusion. *AI* artificial intelligence.

**Figure 2:**
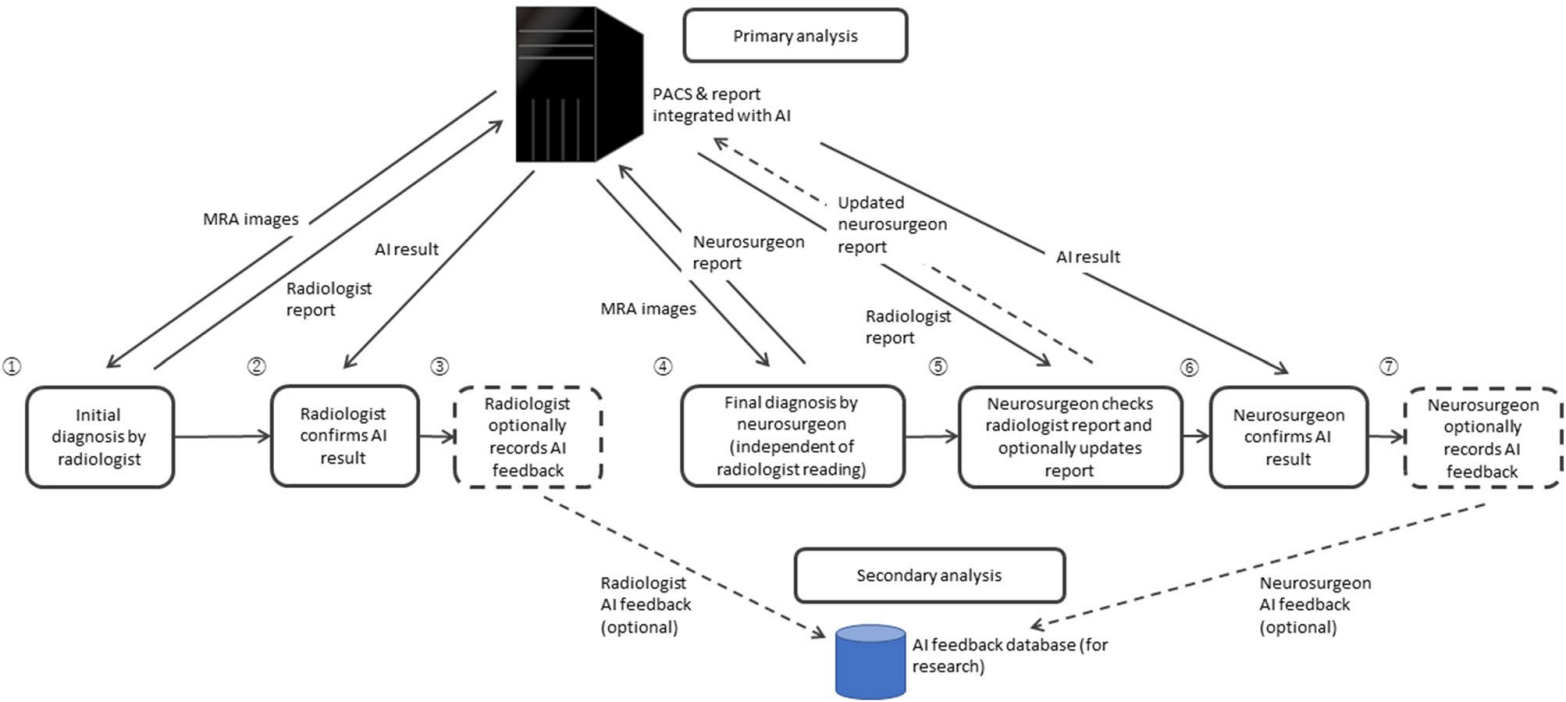
Overview of the reading process. *AI* artificial intelligence, *MRA* magnetic resonance angiography, *PACS* picture archiving and communication system.

A total of 10,000 MRI and MRA images from participants who underwent Brain Dock examinations between December 2018 and July 2019 using the remote imaging system were analyzed, excluding cases with poor image quality or those who stopped the examination because of feeling sick during the process (Fig. 1). In addition, no cases of post-craniotomy or having clips or other surgical instruments in the cranium were included. The age; sex; BMI; medical history, including hypertension, diabetes, dyslipidemia, arrhythmia, dementia, and stroke; surgical history; and the presence or absence of UCAs of all participants were recorded. The presence or absence of a UCA was classified into the following three categories: “definitive,” “none,” and “suspicion.” A “suspicion” case was defined as a case that, in the judgment of the reading physician, could not be completely ruled out as a definitive UCA and was therefore used as a positive case (i.e., presence of UCA) in the analysis. Ground truth determination involved an initial diagnosis by the radiologist, followed by a final diagnosis by the neurosurgeon who reviewed the diagnosis of the radiologist. Both initial and final diagnoses were recorded in the report system. Optionally, both the radiologist and neurosurgeon were allowed to provide detailed feedback about the AI output, labeling each candidate output as either true positive or FP and recording missed UCA (false negative). This information was recorded in the AI feedback database (Fig. 2).

The diagnosis algorithm was tuned on April 7, 2019. The MRI and MRA images of exactly 5000 consecutive participants acquired before and after the tuning of the algorithm were both extracted. The abovementioned examination parameters, along with the results of the final diagnosis of UCA recorded in the report system, were compared and analyzed as the primary analysis. Sensitivity and FPs/case metrics were computed for all 10,000 cases.

Subsequently, a secondary analysis was performed only on the cases for which detailed AI feedback was provided. We extracted 1359 cases where the feedback was given before algorithm tuning and 859 cases where the feedback was given after tuning. The examination parameters and rating of each AI candidate output were again compared and analyzed. The assessment of diagnostic accuracy for UCA diagnosis was specifically based on the comparison of sensitivity and the FP/case.

To ensure highly accurate ground truth with minimal error, all physicians who participated in this study were selected from doctors with more than 10 years of experience and were all board-certified radiologists of the Japan Radiological Society or board-certified neurosurgeons of the Japan Neurosurgical Society. There were more than 7000 board-certified neurosurgeons in Japan in 2018, who could also diagnose MRI findings on Brain Dock like neuroradiologists in Europe or the United States of America. Therefore, in this study, neurosurgeons were fully qualified to perform the final diagnosis.

### Algorithm development

The training external dataset for UCAs and the detection algorithm used in this study have been described previously^12^. The UCA detection algorithm consisted of a processing pipeline based on a convolutional neural network (CNN), which was trained from scratch on several thousand three-dimensional (3D) patches extracted from hundreds of time-of-flight MRA source images. The CNN was used to classify each 3D patch as either a non-aneurysm or an aneurysm.

**Figure 3:**
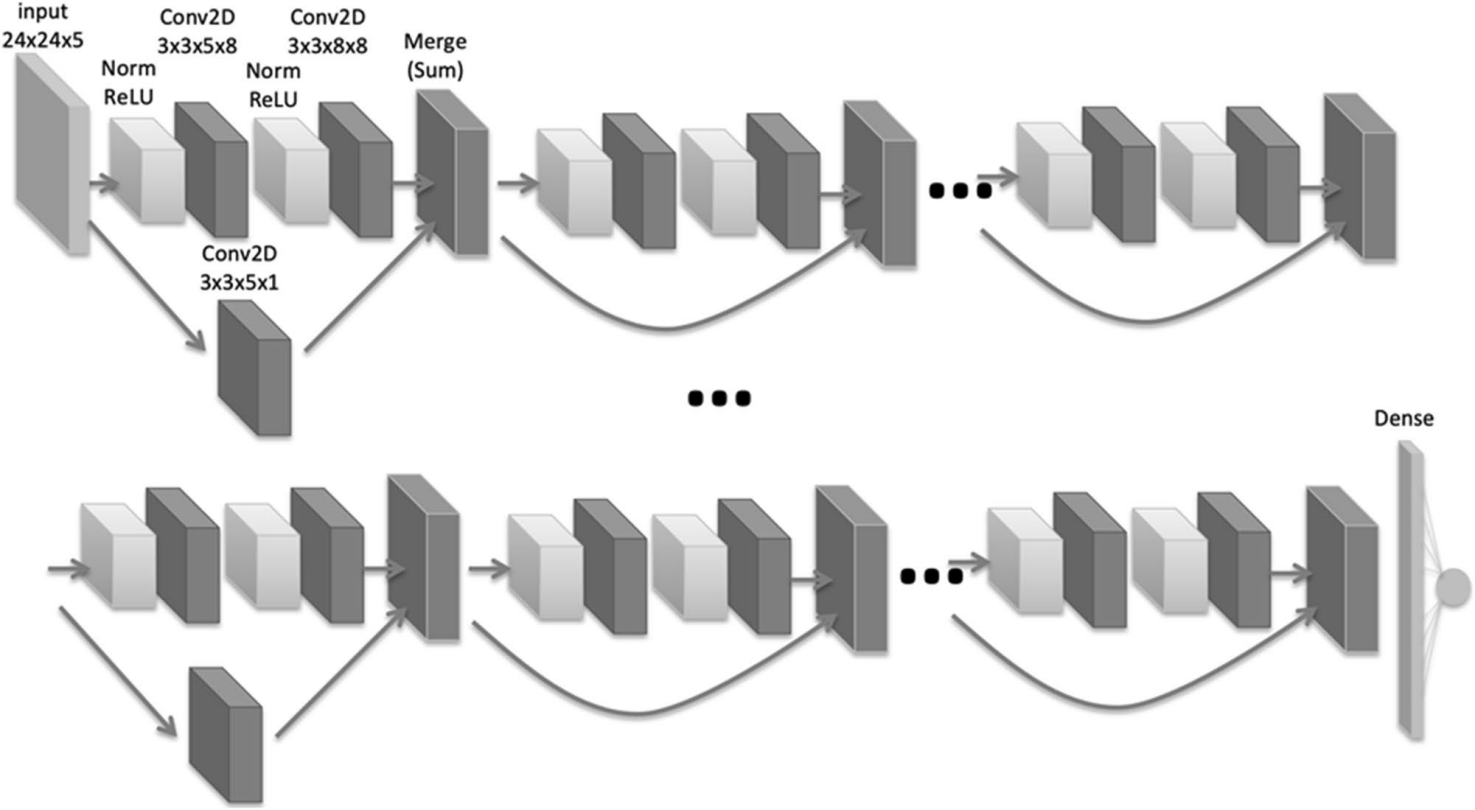
ResNet-18 convolutional neural network consisting of 18 blocks of convolutional layers, average pooling with batch normalization concatenated with a convolutional skip connection, and one fully connected layer. The activation function used is a rectified linear unit. Conv2D, 2D convolution layer; dense, dense (fully connected) layer; merge, merge (sum) layer; norm, batch normalization layer; *ReLU* rectified linear unit.

**Figure 4:**
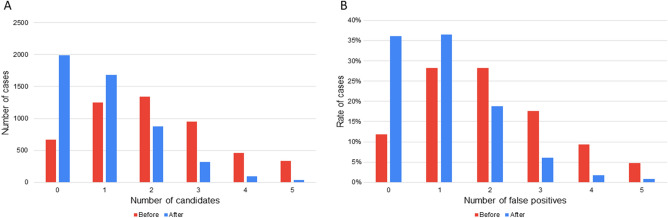
(**A**) Number of cases by the number of candidates before and after tuning the artificial intelligence (AI) algorithm in the primary analysis. The number of cases is the highest with 0 or 1 candidate and is negligible with 4 or 5 candidates after tuning the AI algorithm. (**B**) Rate of cases by the number of false positives (FPs) before and after tuning the AI algorithm in the secondary analysis. The number of cases is the highest with 0 or 1 FP and is negligible with 4 or 5 FPs after tuning the AI algorithm.

**Figure 5:**
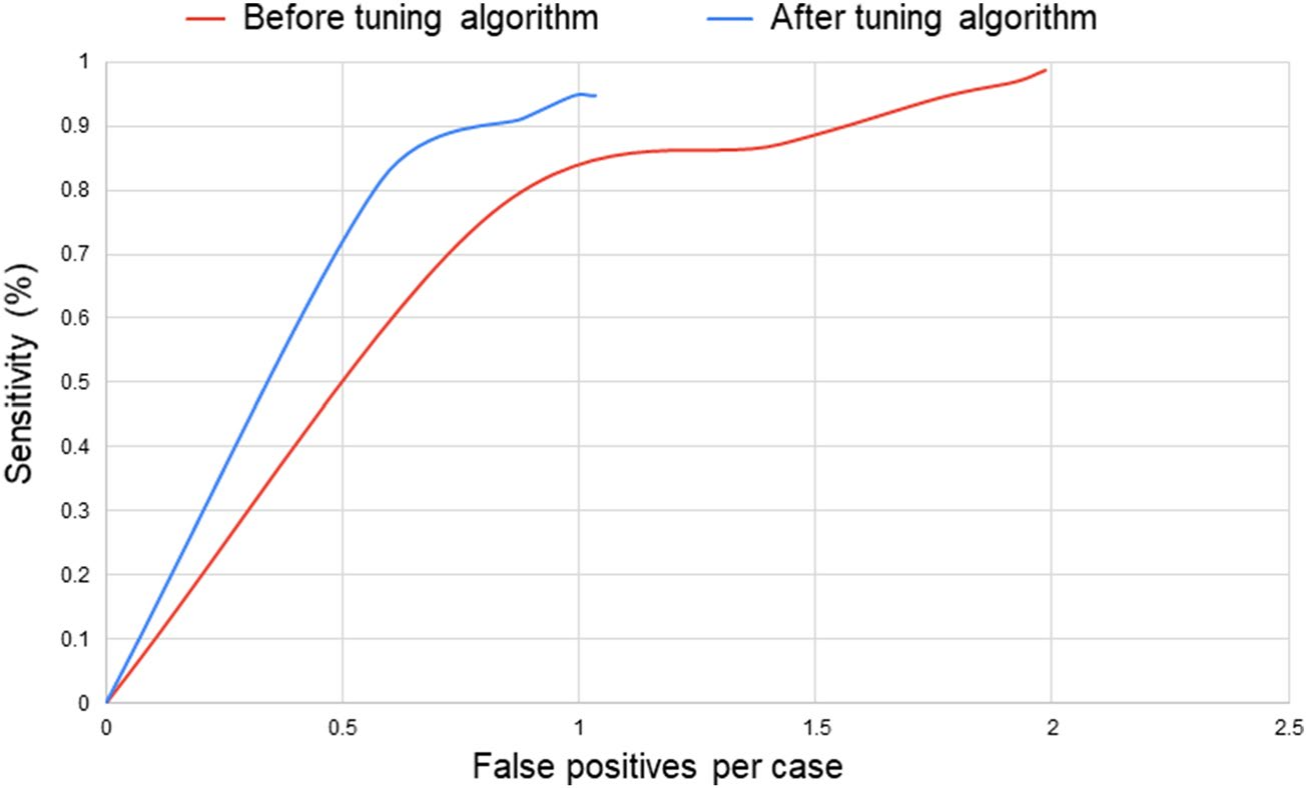
The relationships between the sensitivity and false positives/case before and after tuning the AI algorithm are shown with red and blue lines, respectively, in the secondary analysis.

The algorithm pipeline was as follows: vessel extraction was performed using a thresholding method, the 3D volume was resampled to isovoxel, and the principal curvature coefficient was used for extracting key points^24^. Candidate 3D patches were then extracted around each key point and fed into the CNN. The CNN was a ResNet-18^25^ composed of 39 convolutional, 1 average pooling, and 1 fully connected layers (Fig. 3), considering inputs of small 3D volumes composed of five slices of 24 × 24-pixel patches and outputs of 0 (non-aneurysm) to 1 (aneurysm) scores. The CNN was coded in Python using the Keras (Tensorflow) library and trained for 100 epochs using the following hyperparameters: learning rate = 0.002, beta_1 = 0.9, beta_2 = 0.999, epsilon = 0.00000001, and schedule decay = 0.004. All key points with scores below a predefined threshold were removed, and nearby key points were subsequently grouped through a clustering process. For each cluster, the center of gravity of the comprising key points was calculated and used as the coordinate of the final output of the algorithm. We evaluated the results of tuning a parameter in the postprocessing part of the algorithm. When the output of the CNN was close to 1, the algorithm judged that the probability of the candidate being a true aneurysm was higher. We evaluated the effect of excluding candidates based on the CNN output at two different thresholds. The cutoff threshold, which was previously set to 0.5, was increased to 0.8 after April 7, 2019 (Figs. 4 and 5).

Any candidate whose CNN output score was lower than the threshold was rejected and only the remainder were presented to the user.

### Statistical analysis

All analyses were conducted using R software (version 3.1.2, http://www.r-project.org/). The chi-square test was performed using background factors of the participants, such as age; sex; and medical history, including hypertension, diabetes, dyslipidemia, arrhythmia, cerebrovascular disorder, and surgical history. *P* < 0.05 was considered statistically significant.

In the primary and secondary analyses, we performed Student’s *t*-tests for FPs/case and *z*-tests for sensitivity before and after tuning. FPs/case was computed as the total number of FPs divided by the total number of cases. It should be noted that this number can be higher than 1.0, since the AI can output multiple candidates per case. We plotted a histogram showing the distribution of cases for each number of FP output by the algorithm, compared before and after tuning the algorithm. The relationship between the sensitivity and the FPs/case of UCA detection following the changes in the candidate output points in each group was graphed and compared before and after tuning the AI diagnosis algorithm. The curves were obtained by limiting the number of candidates output by the algorithm to 1, 2, 3, 4, and 5 and computing both the sensitivity and average number of FPs/case in each case, resulting in 5 points for each curve.

### Ethics approval

This study was approved by the appropriate institutional review board (approval number E-1762).

### Informed consent

Individual data were anonymized and collected during routine Brain Dock examinatin; the requirement for individual informed consent was thus waived.

## Data Availability

The anonymized data that support the findings of this study are available from the corresponding author upon reasonable request.
